# Fisetin Suppresses the Proliferative and Migratory Behavior of HeLa Cells by Modulating Aberrant Epigenetic Marks (Writers and Erasers)

**DOI:** 10.3390/epigenomes10010003

**Published:** 2026-01-12

**Authors:** Nazia Afroze, Reham I. Alagal, Lujain A. Almousa, Ritu Raina, Prathap Bava, Lizna Mohamed Ali, Tarique Noorul Hasan, Arif Hussain

**Affiliations:** 1 School of Life Sciences, Manipal Academy of Higher Education, Dubai P.O. Box 345050, United Arab Emirates; afroze.nazia@gmail.com (N.A.); rituraina123456@gmail.com (R.R.); prathap.bava@manipaldubai.com (P.B.); 2 Department of Health Sciences, College of Health and Rehabilitation Sciences, Princess Nourah Bint Abdulrahman University, P.O. Box 84428, Riyadh 11671, Saudi Arabia; rialagal@pnu.edu.sa (R.I.A.); laalmousa@pnu.edu.sa (L.A.A.); 3 Department of Microbiology and Immunology, College of Medicine and Health Sciences, United Arab Emirates University, Al Ain P.O. Box 17666, United Arab Emirates; lizna@uaeu.ac.ae; 4 Department of Molecular Genetics, PureLab, Sh. Tahnoon Bin Mohammed Medical City (STMC), Al Ain P.O. Box 134808, United Arab Emirates; tarique.noorulhasan@purelab.ae

**Keywords:** fisetin, epigenetic modification, DNA methylation, epigenome, methyl sequencing

## Abstract

Purpose: The reversible deviant in epigenomic modulations is the highlight of developing new anti-cancer drugs, necessitating the use of fisetin as an epigenetic modifier in the study. Methods: In silico and molecular studies were performed to analyze the modulatory effect of fisetin on various writers and erasers. Further, whole genome DNA methylation sequencing and expression studies were performed. Global DNA methylation-LINE 1 kit was used to check global DNA methylation. Additionally, the effect of fisetin on migration was evaluated by colony, scratch, and invasion assays and qPCR and protein expression studies of migration-related genes were carried out on HeLa cells. Results: In silico studies have supported that fisetin interacts with writers and erasers in their catalytic site and the simulation studies showed minimum fluctuations in energy and temperature over a 10 ns timescale indicating that these complexes are likely to remain stable. Fisetin (20–50 µM) dose-dependently inhibited DNA methyltransferases (DNMT), histone deacetyl transferases (HDAC), histone acetyl transferases (HAT), and histone methyltransferases (HMT) activities at 48 h, with inhibition ranging from 24 to 72% compared to the control. The expression and enzymatic activity of these proteins, along with various H4 and H3 modification marks, were observed to be altered following fisetin treatment at 48 h. Fisetin treatment reduced promoter methylation in various tumor suppressor genes ranging from 15.29% to 76.23% and leading to the corresponding reactivation of important tumor suppressor genes; however, it did not lead to any alteration in the global DNA methylation compared to untreated controls linked with the anti-migratory properties of fisetin as the percentage of migrated cells dropped from ~40% to ~8%. Conclusions: This study gives a mechanistic insight of fisetin as a potential epigenetic modifier in HeLa cells.

## 1. Introduction

Genome-wide epigenetic alterations are characterized by global DNA hypomethylation, promoter-specific CpG island hypermethylation, and diverse histone modifications such as methylation, phosphorylation, acetylation, etc., and might be responsible for heterogeneity in tumor cells. Epigenetic changes are mediated by different classes of enzymes, such as DNA methyltransferases (DNMTs) and demethylases that work on DNA, as well as several histone-modifying enzymes including deacetylases (HDACs), acetyltransferases (HATs), methyltransferases (HMTs), and phosphorylases, etc. [1,2]. As these epigenetic changes are reversible, they provide a valuable opportunity for improving targeted therapies that can modulate cancer-related gene expression. In the process of cancer development, various DNMTs (DNMT1, DNMT3A, and DNMT3B) [3] and HDACs (HDAC 1, 2, 3, and 6) are reported to be overexpressed in different cancers [4,5]. Overexpression of HDAC levels, along with disturbed epigenetic changes such as DNA methylation and histone modification, contribute to the silencing of tumor suppressor genes [6].

Promoter hypermethylation is a primary mechanism of gene silencing at tumor suppressor genes (TSGs), either through increased expression of DNMTs or by the recruitment of methylated DNA-binding proteins [6,7]. The promoter region is documented to be hypermethylated in multiple cancer types, including genes such as p16INK4a, which regulates the cell cycle, and hMLH1, which is frequently silenced in colorectal carcinoma [8]. Other tumor suppressor genes, including WIF1, RUNX1, RARβ, p53, DAPK, etc., have also been shown to undergo promoter hypermethylation in several cancers especially in cervical cancer. Interestingly, histone modifications are mediated by various histone writers and erasers such as HATs, HMTs, HDACs, and KDMs (histone lysine demethylases) can activate or repress the expression of genes that consequently may propel the cells towards carcinogenesis [9]. HMTs add 1–3 methyl (-CH_3_) groups to specific lysine residues, depending on the lysine residue involved, these marks may lead to transcriptional activation or repression. Several lysine methylation patterns on histone H3/H4 (H3K27, H3K9, and H4K20) are correlated with transcription repression, whereas H3K4, H3K36, and H3K79 are responsible for transcription activation. DNA methylation silenced promoters show loss of H3K4 and an increase in H3K9 methylation with a subsequent increase in deacetylation that leads to silencing [10,11]. The serious side effects of different DNMT and HDAC inhibitors (decitabine, Trichostatin, etc.), which are currently under use for the treatment of cancer patients, as well as the unsafe cytotoxic profile and non-specificity of the drugs warrants a quest for safer drug candidates [12]. It is encouraging to know that a variety of bioactive phytochemicals can modulate several epigenetic enzymes such as HDAC, HAT, DNMT, and HMT [13] that may consequently alter the expression of important tumor suppressor, oncogene, and tumor promoter genes, etc. Polyphenols like Quercetin, chrysin, EGCG, myricetin, etc., are confirmed to hinder activities of HDAC and DNMT, with the subsequent reactivation of TSG [5]. The phytochemical, fisetin (3,7,3′,4′-tetrahydroxyflavone), used in this study has been shown to exhibit anti-oxidant, anti-neoplastic, anti-inflammatory, and anti-invasive properties [14]. Research on fisetin’s effects on the epigenome is just beginning to emerge. One study reported the inhibition of DNMT and HAT by fisetin [15]. Further, an in silico study has reported that fisetin also acts as HDAC 3, 4, and 6 inhibitors [16]. This study aims to first investigate the in silico effect of fisetin on the activity of various writers and erasers followed by analysis of the activities and expression of different chromatin modification enzymes. Further, the study intends to examine the anti-migratory potential of fisetin through cell-based and molecular studies and finally to analyze the promoter methylation status of TSGs after fisetin treatment in HeLa cells.

## 2. Results

### 2.1. Molecular Docking of Fisetin with Epigenetic Enzymatic Proteins

**Figure 1:**
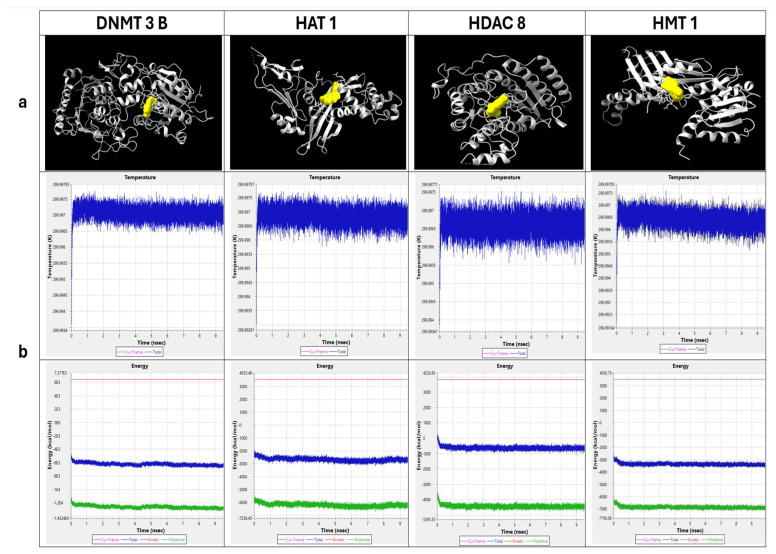
In silico analysis: (**a**) Epigenetic enzymatic proteins docked with Fisetin viewed in ChimeraX. (**b**) Molecular dynamics simulation of epi-enzymes and fisetin complex Temperature–Time graph indicates blue color spikes as the system temperature whereas Energy–Time graph indicates blue and green color as total energy & potential energy respectively.

Docking studies were conducted to assess fisetin interaction with key epigenetic enzymes. Using CB0-Dock2, fisetin was docked with several epigenetic proteins, including DNMT3B, HAT1, HMT1, and HDAC8, to understand its potential molecular interactions (Figure 1a). The binding pocket with the lowest Vina score was chosen, and the interacting coordinates were noted, along with ligand binding to specific amino acids (Figure 1a, Table 1). Simulation studies showed that the binding of fisetin with epi-enzyme complexes was stable, as indicated by minimum fluctuations in energy and temperature over a 10 ns timescale. Thus, these complexes are likely to remain stable under physiological conditions (Figure 1b).

### 2.2. PLIP Analysis Indicated the Interacting Bond Between Epi-Enzymes and Fisetin/Native Inhibitor Complex Led by Energy Minimization Comparison

The interacting amino acids for Fisetin–protein_complex.pdb and Nativeinhibitor–protein_complex.pdb were identified and summarized (Table 1). In this study, DNMT3B, HAT1, HMT1, and HDAC8 were taken; the interacting amino acids and hydrogen bonding patterns were largely similar between the fisetin and native inhibitor complexes. The analysis shows that fisetin binds to the proteins similarly to the native inhibitor. This suggests that fisetin could potentially mimic the action of the native inhibitor, making it a promising candidate for further study as a modulator of these epigenetic enzymes. Although HMT1 did not share the same docking site with fisetin compared to the native inhibitor, it formed multiple hydrogen bonds and interacted with several amino acids, which shows that binding similarity is not the only factor that determines a stable interaction. Even when bound in different pockets or orientations, the network of non-covalent and hydrogen bonds can stabilize the ligand within the protein, allowing it to remain firmly bound and functionally relevant.

The SwissPDB Viewer (SPDBV) was used to analyze the total energy minimization of docked complexes (fisetin/native inhibitor and epi-enzymes). The energy minimization analysis revealed that the protein–fisetin complexes exhibited total energy values very close to those of the protein–native inhibitor complexes. In DNMT3B, HAT1, HMT1, and HDAC8, the total energy was observed and compared between fisetin and native inhibitor complexes (−30,962.518 to −30,970.602; −17,527.574 to −17,553.438; −17,683.092 to −17,689.406; and −13,636.612 to −13,631.291, respectively), as these complexes showed minimal differences in total energy between fisetin and the native inhibitor. These small variations suggest that fisetin can stabilize the protein structures in a manner comparable to the native inhibitor. Thus, fisetin demonstrates similar binding properties and potential inhibitory effects against these epigenetic proteins.

### 2.3. Fisetin Modulated the Activity of Dnmt, Hdac, Hat, and Hmt That Methylate H3 at K27

**Figure 2:**
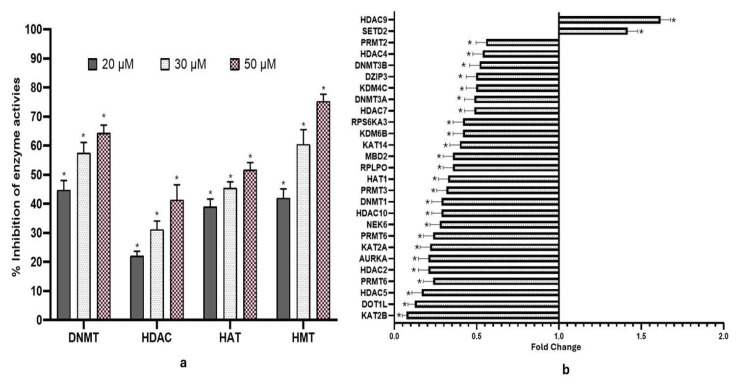
(**a**) Bar graph indicating % inhibition of DNMT, HDAC, HAT, and HMT enzyme activities followed by fisetin treatment at 48 h. The data is shown as the mean ± standard deviation of three independent experiments. Two-Way ANOVA * = *p* < 0.05. (**b**) Fisetin treatment (50 µM, 48 h) altered the expression of various chromatin modification enzymes compared to the untreated control.

DNMT and HDAC activities were reduced significantly in a concentration-dependent manner following fisetin treatment relative to controls. Compared to DMSO control, DNMT activity reduced to 48.4%, 51.4%, and 63.2% at 20, 30, and 50 µM at 48 h, respectively, in treated samples (Figure 2a), while fisetin at 20, 30, and 50 µM reduced the HDAC activities by 24%, 30%, and 40%, respectively (Figure 2a). Similarly, fisetin treatment also reduced histone acetyltransferase (HAT) activity, and it was observed to be reduced by 38%, 45%, and 49%, respectively (Figure 2a). The HMT that methylates H3 at K27 activity was inhibited by fisetin (20, 30, and 50 µM), and inhibition increased in a concentration-dependent manner by 41%, 61%, and 72%, respectively, compared with DMSO control (Figure 2a). Data are presented as the mean ± standard deviation of three independent experiments. Two-way ANOVA * *p* < 0.05.

### 2.4. Fisetin Modified the Expression of Chromatin-Modifying Enzymes

Fisetin downregulated various chromatin-modifying enzyme expressions including DNA methyltransferases, DNMT1, DNMT3A, and DNMT3B; histone deacetylases such as HDAC2, HDAC4, HDAC5, HDAC7, HDAC9, and HDAC10; and HAT1 (histone acetyltransferase) (Figure 2b). HeLa cells treated with 50 µM fisetin for 48 h resulted in downregulation of several chromatin-modifying enzymes, including acetyltransferases (KAT2A, KAT5, KAT14), phosphorylases (AURKA, AURKB), ESCO1, NEK6, DOT1L, and the lysine demethylase KDM6B, while the expression of HDAC9 and SETD2 were increased.

### 2.5. Fisetin Reduced the Proliferative and Migratory Ability of HeLa Cells at Both the Cellular and Molecular Level

**Figure 3:**
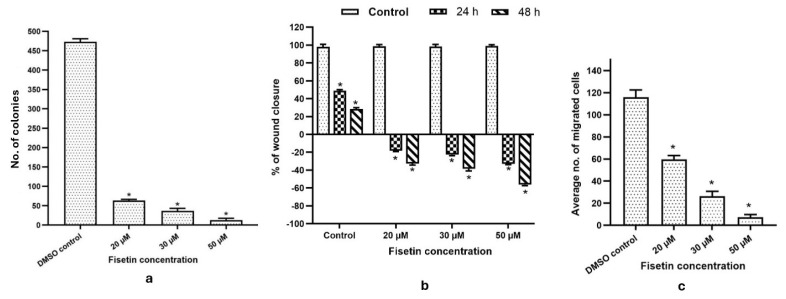
(**a**) The bar graph illustrates the number of colonies in HeLa cells grown for two weeks under DMSO control and fisetin treatment at 20, 30, and 50 µM. (**b**) Bar graph showing concentration-dependent increase in wound width. (**c**) Invasion assay showing reduced invasive capability of the treated cells compared to control on increasing fisetin doses (20, 30, and 50 µM). * *p* > 0.05.

Treatment with fisetin at 20, 30, and 50 µM led to a dose-dependent decrease in the number and size of HeLa cell colonies, highlighting its anti-proliferative effect. While control cells had a survival factor of ~95%, colony counts dropped to 90%, 73%, and <10% at 20, 30, and 50 µM, respectively (Figure 3a). These observations highlight fisetin’s cytostatic and anti-migratory activities. Treatment with 20–50 µM fisetin delayed wound closure, with remaining wound width increasing from 18% to ~25% at 24 h and from 21% to 43% at 48 h, whereas control cells treated with DMSO achieved complete closure by 72 h (Figure 3b). The anti-migratory properties of fisetin were evident, as the percentage of migrated cells dropped from ~40% to ~8% at 20–50 µM, compared with DMSO-treated controls (Figure 3c).

**Figure 4:**
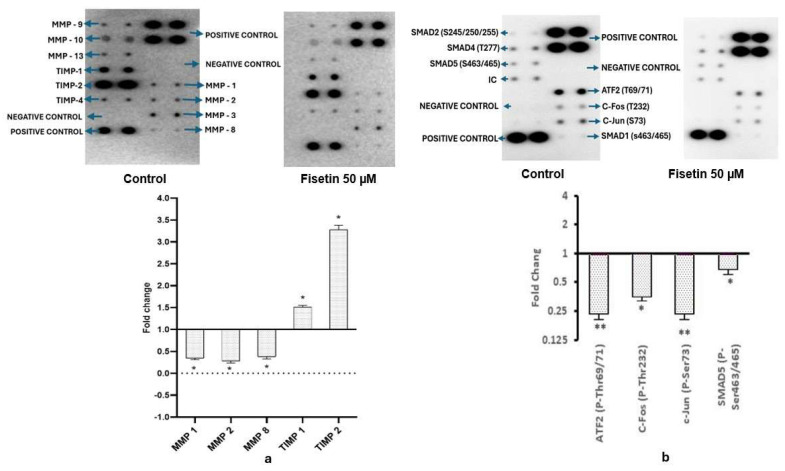
(**a**) Images of nitrocellulose membranes, along with accompanying graphs, demonstrate reduced expression of several MMPs and increased TIMP expression after 48 h of fisetin treatment, compared with control cells. (**b**) Images of nitrocellulose membranes and corresponding bar graphs depict changes in the fold expression of phosphorylated proteins involved in TGF-β signaling following treatment with 50 µM fisetin for 48 h, compared with control cells. * *p* > 0.005 & ** *p* > 0.001.

**Figure 5:**
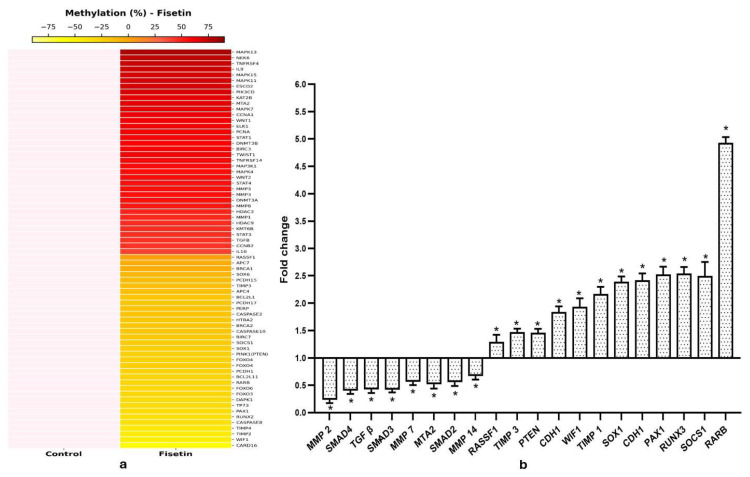
(**a**) Differential methylation of various genes between control and 20 µM fisetin-treated samples (48 h) is shown as a heatmap representing fold changes in methylation percentage. (**b**) Bar graphs summarize qPCR results, showing that fisetin treatment enhanced the expression of tumor suppressor genes and suppressed multiple oncogenes that took part in cell migration relative to DMSO-treated HeLa cells (* *p* < 0.001).

Fisetin was found to suppress WNT/TGFβ pathway at transcript level by reducing the manifestation of WNT1, WNT2, SMAD1, SMAD2, SMAD3, SMAD4, and TGFβ1 transcripts along with genes involved in invasion and migration such as MMP2 (FC; 0.24), MMP7 (FC; 0.57), MMP14 (FC; 0.6), MTA2 (FC; 0.55), and TWIST while upregulating TIMP1 (FC; 2.1) and TIMP3 (FC; 1.4) (Figure 5b). Consistently, at the protein level, expressions of MMP1 (FC; 0.37), MMP2 (FC; 0.31), and MMP8 (FC; 0.34) were downregulated by fisetin. Fisetin also upregulated TIMP1 (FC; 2.1) and TIMP3 (FC; 1.4) expressions (Figure 4a). Moreover, fisetin was also observed to downregulate the expression of different phosphorylated proteins associated with WNT/TGFβ/SMAD pathway, such as ATF2 (P-Thr69/71) (FC; 0.2395), c-Fos (P-Thr232) (FC; 0.39), c-Jun (P-Ser73) (FC; 0.63), and SMAD5 (P-Ser463/465) (FC; 0.67). The relative expression of protein was performed against the DMSO control (Figure 4b).

### 2.6. Fisetin Altered the Promoter Methylation of Various Genes, but Line 1 Global DNA Methylation Remains Unaffected

To evaluate fisetin’s effect on DNA methylation, treated and control samples were analyzed for methylation differences. Each sample was run in duplicate, and the resulting data files were combined to identify differentially methylated regions (DMRs), with the average of duplicates used for analysis. A 10% cutoff was applied to define DMRs and differential variations in methylation patterns between control and fisetin-treated (20 µM) HeLa cells were further illustrated using a heatmap generated with the R package gplots (Figure 5a) showing differential variation in methylation at promoter regions for the top 70 genes between control and fisetin-treated samples.

As evident from methyl-sequencing data, fisetin treatment causes hypomethylation and reactivation of various TSGs such as TP53 (55.2%), PAX2 (76.23%), PAX1 (58.53%), PAX6 (28.3%), PTEN (PINK1) (47.85%), RASSF1 (15.29%), WIF1 (73.45%), VHL (41%), DAPK1 (54.59%), RUNX1 (37.6%), RUNX2 (70.6%), RARβ (52.89%), SOCS1 (43.75%), SOX1 (46.7%), FOXO3 (58%), PERP (49.3%), TIMP3 (35%), etc., whereas genes such as TGFβ (36.5%), MMP1 (39.92%), MMP2 (74.2%), MMP3 (48.6%), MMP8 (45.52%), MMP20 (55%), and MTA2 (63%) were hypermethylated (Figure 5a).

DNA from cells treated with 20, 30, or 50 µM fisetin exhibited no significant differences in LINE-1 methylation compared to untreated controls, highlighting the selective action of fisetin.

### 2.7. Hypo- and Hypermethylation Correlate Well with RNA Expression in Fisetin-Treated HeLa Cells

To validate that fisetin enhanced TSG expression through promoter demethylation, selected genes were analyzed using qRT-PCR. Fisetin treatment raised the expression of RASSF1, VHL, TP53, PTEN, WIF1, FHIT, PAX1, SOX1, SOCS1, TIMP1, TIMP3, RUNX3, RARβ, and DAPK1, corresponding to promoter hypomethylation as evident through methyl-seq results (Figure 5b).

### 2.8. Fisetin Altered Methylation and Acetylation Marks on Histone H3 and H4

Treatment with fisetin significantly decreased methylation marks on histones H3 and H4. When cells were exposed to 50 μM fisetin for 48 h, we observed inhibition of all methylation types (mono-, bi-, and trimethylation) at multiple H3 positions, including K4 (Lysine 4), K9 (Lysine 9), K27 (Lysine 27), K36, and K79 (where mono- and trimethylation were specifically reduced). Acetylation marks were similarly affected—H3K9ac, H4K14ac, H4K18ac, and H3K56ac on histones 3 and 4 all showed reduced levels. We also found that phosphorylation marks like H4Ser10ph, H3Ser28ph, H4R3m2a, and H4R3m2s decreased after fisetin treatment at 50 μM (Table 2).

## 3. Discussion

The dysregulation of cellular signaling pathways and regulatory genes owing to genetic and epigenetic modification often leads to tumorigenesis. An aberrant alteration of epigenome may repress regulatory genes involved in cell growth, cell death, etc., through promoter hypermethylation and/or activation of genes linked with invasion and migration of cancer cells through hypomethylation and global DNA hypomethylation, which subsequently drives the cells toward carcinogenesis [17,18]. Aberrant methylation patterns and enhanced expression of DNMTs have been reported in multiple malignancies [3].

Furthermore, epigenomic equilibrium is maintained by the synergetic action of epigenetic enzymes, and any deregulation in the expression of these enzymes such as HATs, HMTs, HDACs, HDMs, histone phosphorylases, and ubiquitinases that are responsible for acetylation, methylation, deacetylation, demethylation, phosphorylation, and ubiquitination, respectively, will alter the gene accessibility [9]. Hence, these histone modifiers could be targeted for the better diagnosis, treatment, and prognosis of cancer [9]. The epi-drugs in use, such as decitabine (a DNMT inhibitor) and Trichostatin A (HDAC inhibitor), against breast cancer, lung cancer, etc., show lack of specificity and serious side effects; therefore, a quest for safer drug candidates is warranted [9,12]. It is encouraging to know that a variety of bioactive phytochemicals can alter the expression of important tumor suppressor, tumor promoter, and oncogene genes via modifying chromatin modification and DNA methylation as they can modulate different enzymes like HDAC, HAT, DNMTs, and HMT [13,19,20,21,22]. Polyphenols like quercetin, EGCG, chrysin, and myricetin are established inhibitors of HDAC and DNMT enzymes, resulting in the reactivation of tumor suppressor genes [5,8]. In comparison, studies on fisetin’s epigenetic modulation are only beginning to emerge. Therefore, this study aimed to explore the effects of fisetin on the activity and expression of various epigenetic enzymes at both in silico and molecular levels, as well as to examine the promoter methylation status of tumor suppressor genes (TSGs) associated with multiple cancer hallmarks in HeLa cells.

Molecular docking studies between fisetin and epi-enzymes (Figure 1a) showed significant interactions at the catalytic site of these enzymes, and the interacting amino acids and hydrogen bonding patterns between fisetin and DNMT3B, HAT1, and HDAC8 are very similar to those of the native inhibitor complexes. The results also showed that fisetin binds to the proteins in a manner very similar to the native inhibitor (Table 1). Further, the molecular dynamics simulation study showed that energy and temperature fluctuations were minimal, enabling the complex to remain stable even under physiological conditions (Figure 1b); hence, it is apparent that fisetin can mimic the action of the native inhibitor, making it a promising candidate as an epigenetic modifier. Earlier studies have reported the inhibition of DNMT and HAT by fisetin [15]. Further, an in silico study has reported that fisetin also acts as HDAC 3, 4, and 6 inhibitors [16]. Although HMT1 does not share identical docking sites with fisetin compared to the native inhibitor, it forms multiple hydrogen bonds and engages with several amino acids (Table 1). This shows that binding similarity is not the only factor that determines stable interaction. Even in different pockets or orientations, the network of hydrogen bonds and non-covalent interactions can stabilize the ligand within the protein, allowing it to remain firmly bound and functionally relevant. At the molecular level, fisetin was observed to downregulate various chromatin-modifying enzymes (writers and erasers) such as acetyltransferases (HAT1), deacetylases (HDAC2, 4, 5, 7, and 10), phosphorylases at the transcript level, etc. (Figure 2b). Altered expression of these enzymes has shown subsequent repercussions. An enhanced DNMT expression causes hypermethylation of 5′ promoter CpG islands, resulting in repressed gene expression in cervical carcinoma [3,23]. Interestingly, fisetin treatment significantly reduced DNMT1, 3A, and 3B at the transcript level to an FC ≤ 0.5. This was consistent with reduced DNMT activity at the biochemical level, as fisetin inhibited DNMTs in a dose-dependent manner compared to the control (Figure 2a). PI3K/AKT and WNT pathways are responsible for stabilizing DNMT1 proteins and hence aid in its activity [24]. Fisetin has been shown to repress WNT and PI3K/AKT pathways and, therefore, indirectly affects DNMT activity. Fisetin’s impact on the reduced activity and expression of DNMT enzymes may result in reactivation of TSGs that would exert anti-proliferative and apoptosis-inducing effects [23,25]. Polyphenol-mediated reduced expression of DNMTs has been reported in various in vitro and in vivo carcinoma models [4,5].

Histone acetylation and deacetylation are key modulators of carcinogenesis. HDACs also deacetylate non-histone proteins such as TP53, leading to loss of function [8]. The overexpression of HDAC1, HDAC2, HDAC3, and HDAC6, together with aberrant DNA methylation, contributes to tumor suppressor gene silencing [8]. Fisetin effectively inhibited HDAC activity, reducing the expression of HDAC2, HDAC4, HDAC7, and HDAC10, while increasing HDAC9 levels (Figure 2a,b). This downregulation reactivated tumor suppressor genes and suppressed cancer cell growth [9], highlighting fisetin’s potential as an epigenetic modulator in cancer therapy.

HAT1, a key enzyme essential for clonogenicity and frequently upregulated in cancers such as HeLa [26], was significantly downregulated by fisetin, accompanied by a dose-dependent reduction in overall HAT activity (Figure 2a,b). This suppression supports fisetin’s anti-proliferative potential. Moreover, fisetin markedly reduced KAT2A expression (an oncogenic protein) and diminished H3K9 mono-, di-, and trimethylation levels [27]. Correspondingly, decreased acetylation at H4K5, H4K12, and H4K16 was observed, consistent with reduced HAT activity at both biochemical and transcript levels (Table 2, Figure 2a,b). Similar effects of polyphenols have been reported in various cell lines, including HeLa [8]. Additionally, fisetin treatment lowered H4K20 methylation (mono-, di-, and trimethylation) and H4Ser1 phosphorylation at 50 µM concentration (Table 2).

The number (mono-, di-, or trimethylation) and position of methyl groups being added on histone H3 and H4 proteins by histone methyltransferases (HMTs) result in transcriptional activation or repression; for example, methylation on H3K27 leads to the silencing of different TSGs [28]. Notably, fisetin reduced HMT activity, accompanied by the suppression of these histone H3 marks (Figure 2a) (Table 2). Similarly, chrysin has been reported to decrease these histone H3 marks in a manner comparable to fisetin [8]. The phosphorylation of histones by kinases influences a number of key processes in the cell, including how genes are transcribed, whether apoptosis occurs, how DNA is repaired, and the condensation of chromatin that happens when cells divide [29]. Such phosphorylation events modify protein interactions and recruit transcriptional complexes [29]. Oncogenic kinases and methyltransferases including AURKA, AURKB, ESCO1, NEK6, and PRMTs that promote carcinogenesis are notably upregulated in cervical cancer [5]. Fisetin significantly downregulated these genes, with reduced NEK6 expression promoting apoptosis [30]. DOT1L/KMT4, involved in cell growth and angiogenesis, was also suppressed (Figure 2b). Fisetin-mediated reduction of ESCO1 expression may contribute to its induction of DNA damage, cell cycle arrest, and apoptosis (FC ≥ 0.6) (Figure 2b). Moreover, PRMT6, which methylates Fisetin, also downregulated PRMT1, PRMT3, PRMT5, and PRMT6 (Figure 2b). These observations are consistent with prior findings in human osteosarcoma cells, as reported previously [31]. In vitro, fisetin at 20, 30, and 50 µM effectively suppressed HeLa cell migration and invasion, with effects increasing in a concentration- and time-dependent manner (Figure 3b,c). Likewise, fisetin treatment reduced the number of colonies from 95% plating efficiency to only around 30 colonies (Figure 3a). Various polyphenols have been reported earlier with similar results [32]. The epithelial–mesenchymal transition (EMT) is explained as a loss of cell adhesion mediated by proteins such as E-cadherin, vimentin, fibronectin, SNAI1, MTA1, MTA2, TWIST, and MMPs [5]. E-cadherin (CDH1), a major adhesion molecule, suppresses invasion and metastasis, while its inhibition by SNAI1 promotes migration [5]. TGFβ/SMAD signaling drives mesenchymal transition, and its inhibition suppresses MMP and TWIST1 expression [4]. ROBO/TIMPs counteract MMP activity to preserve adhesion [33], whereas TIMP1-3 inactivation and TWIST1 overexpression via WNT signaling enhance metastasis [33]. Fisetin treatment (50 µM) markedly reduced WNT2, SMAD2, SMAD3, SMAD4, and TGFβ1 expression, accompanied by the downregulation of MMP2, MMP7, MMP14, TWIST, and MTA2, and the upregulation of CDH1, TIMP1, and TIMP3, confirming its anti-migratory effect (Figure 4a). At the protein level, fisetin decreased MMP1, MMP2, and MMP8, while increasing TIMP1-2 (Figure 4b). Moreover, fisetin suppressed TGFβ signaling by dephosphorylating ATF2 (P-Thr69/71), c-Fos (P-Thr232), c-Jun (P-Ser73), SMAD4 (P-Thr277), and SMAD5 (P-Ser463/465), leading to reduced AP-1 activity (Figure 4b). This inhibition likely underlies the observed decrease in proliferation and migration as reported previously [34]. This effect was further confirmed by cell-based assays (Figure 3a–c).

Aberrant DNA hypermethylation and chromatin modifications contribute to the silencing of tumor suppressor genes (TSGs) such as BRCA1, p53, p21, p27, RARβ, hMLH1, DAPK, CDH1, p16, APC, MSH2, PTEN, and MLH1 in various cancers [35]. Conversely, oncogenes including c-myc, Ras, CDKs, and cyclins are often upregulated in cervical cancer, correlating with poor prognosis [4,36]. Since epigenetic alterations are reversible, targeting DNA methyltransferases (DNMTs) offers therapeutic potential. Fisetin (20 µM, 48 h) markedly reduced 5′ CpG promoter methylations and reactivated several TSGs, including TP53, PTEN (PINK1), RASSF1, RUNX1, APC2, DAPK1, FOXO3, PERP, RARβ, SOCS1, SOX1, CDH13, TIMP2, TIMP3, WIF1, and VHL, many of which are silenced in cervical cancer (Figure 5a). These genes regulate apoptosis (DAPK1), cell proliferation (PTEN, RARβ), migration and invasion (MMPs, TIMPs), and WNT signaling (WIF1, APC2). Fisetin-induced demethylation restored their expression, verified by qPCR, showing significant upregulation of RARβ, SOX1, and RUNX3 (3–5-fold) (Figure 5b).

The study highlights fisetin’s ability to restore tumor suppressor gene function, an important factor underlying fisetin’s anti-cancer potential in cervical cancer. This study can further be validated under in vivo conditions.

## 4. Material and Methods

### 4.1. In Silico Studies

#### 4.1.1. Ligand Preparation

Using PubChem [37] (https://pubchem.ncbi.nlm.nih.gov/ accessed on 12 September 2025), Fisetin 3D structure is downloaded in SDF 3D format and then, using SMILES formula, the structure (https://cactus.nci.nih.gov/translate/, accessed on 13 September 2025) is converted into .pdb format and saved as ligand (Fisetin.pdb) bioinformatics studies [38]. For visualization the ligand Chimera tool was used [39].

#### 4.1.2. Receptor Preparation

Epigenetic changes alter the chromatin organization and the accessibility of DNA that influences the process of DNA replication, repair, and gene transcription [40]. Epigenetic enzymatic proteins such as DNMT 3B (PDB ID: 6U8V), HAT 1 (PDB ID: 9MJG), HMT 1 (PDB ID: 4HC4), and HDAC 8 (PDB ID: 1T69) were chosen as receptor molecules docked with fisetin (the ligand). The .pdb files were downloaded from RCSB Protein Bank (https://www.rcsb.org/) for the abovementioned epi-enzymes [41]. The downloaded .pdb files were prepared for docking by eliminating metadata, native inhibitors, and water molecules that are bound to receptor molecules and saved as Cleaned.pdb files for further docking studies.

#### 4.1.3. Molecular Docking of Fisetin with Epigenetic Enzymatic Proteins by CB-Dock2

Computer-aided drug discovery (CADD) has become an essential approach for exploring protein–ligand interactions, particularly through techniques like molecular docking [38].

CB-Dock2 is a freely available software, accessible at https://cadd.labshare.cn/cb-dock2/index.php, which utilizes cavity detection-guided blind (auto) docking to predict the binding site and affinities [42].

The ligand (fisetin) was docked with the epigenetic enzymatic proteins (Cleaned.pdb) using auto-blind docking, among the 4 complexes output, the lowest vina score complex protein–ligand docked .pdb file was downloaded (Fisetin–protein_complex.pdb) for further analysis and the docked complex was viewed using ChimeraX, Version 1.11 software [43]. Blind docking was also performed for the native inhibitor with Cleaned.pdb (protein) (Nativeinhibitor–protein_complex.pdb) for further comparative analysis.

#### 4.1.4. PLIP Analysis of Epigenetic Enzymatic Proteins and Ligand (Fisetin/Native Inhibitor) and Energy Minimization by SPDV

The Protein–ligand docked complex .pdb file was uploaded to the freely accessible PLIP analysis tool online (https://plip-tool.biotec.tu-dresden.de/plip-web/plip/index) which indicated the interacting amino acids, hydrogen bonds, and π-stacking between the ligand and protein [44]. Results were compared between Fisetin–protein_complex.pdb vs. Nativeinhibitor–protein_complex.pdb. Further, using SPDV software, all receptor–ligand complexes were energy minimized to determine the total energy minimization of the fisetin docked complex and the native inhibitor complex, and the results were compared.

#### 4.1.5. Molecular Dynamics Simulation Study

A molecular dynamic simulation study was performed by using MyPresto v 5.0 software [45]. This simulation study was performed for the Fisetin–protein_complex.pdb. Initially, global minimization of the complex was performed with 5000 loop limit and generalized Born; after that, global dynamics were simulated with a loop limit of 5,000,000 at 300 K initial constant temperature and energy with a 10 ns time period where all other parameters were kept at default values.

### 4.2. Preparation of Drug Dilutions and Maintenance of Cell Culture

A 69.87 mM fisetin solution (TOCRIS biosciences, Bristol, UK) was prepared using DMSO in serum-free DMEM media. Stock solutions were prepared and stored in aliquots at −20 °C. For experimental treatments, working dilutions were freshly made in the growth medium using the designated model cell line, i.e., HeLa cells, that were purchased from Addexbio (San Diego, CA, USA; Cat No# C0008001). The cell lines were grown in 10% FBS Sigma-Aldrich (St. Louis, MO, USA), -supplemented DMEM media in standard culture conditions at 37 °C with 5% CO_2_.

### 4.3. Analysis of Different Chromatin Modification Enzymes qPCR

After 48 h of treatment with 50 µM fisetin, RNA was isolated from HeLa cells and from corresponding control cells according to the established protocol for analyzing chromatin modification enzymes using the RT^2^ Profiler™ PCR kit Qiagen (Germantown, MD, USA; Cat. No. PAHS-085Z). For qPCR, 1 µg of cDNA was diluted in nuclease-free water, and a 25 µL reaction mixture was assembled by combining the diluted cDNA with the qPCR master mix Qiagen (Germantown, MD, USA; Cat No# 330504) in a 1:1 ratio. The mixture was pipetted into each well containing the pre-coated primers, and the plate was subsequently run on an Applied Biosystems QuantStudio 3 system (Waltham, MA, USA). Using GUSB as a housekeeping gene, the data were normalized, and relative expression levels were calculated as fold changes compared to DMSO-treated controls.

### 4.4. Biochemical Assays

Activity/inhibition assays of DNMT, HDAC, HAT, and HMT H3K27 were performed using EpiQuikTM Nuclear Extraction Kit Epigentek (Farmingdale, NY, USA; Cat. No. OP-0002), HDAC Activity Assay Kit (BioVision, Milpitas, CA, USA; Cat. No. K331), HAT Activity/Inhibition Assay Kit (BioVision, Milpitas, CA, USA; Cat. No. K332), and HMT H3K27 Methyltransferase Activity/Inhibition Quantification Assay Kit (Abcam, Cambridge, UK; Cat. No. ab113454), respectively, following the protocol of the respective kits. A brief protocol of all the mentioned kits was published earlier [46]. *p*-value calculation was performed through two-way ANOVA, and significance was confirmed at *p* ≤ 0.05. Calculation of % enzyme inhibition compared to controls was performed using the following formula:

*Inhibition* % = [1 − *inhibitor sample OD* − *Blank OD/No inhibitor Sample OD* − *Blank OD*] × 100

### 4.5. Impact of Fisetin on Colony Formation, Wound Healing, and Transwell Assays

Colony formation assays assess the competency of a few existing live cells to grow and establish colonies, reflecting their long-term proliferative potential. Cells (~4 × 10^5^ cells/well) were plated and the next day, 20, 30, and 50 µM concentrations of fisetin were used for 48 h, with untreated cells as controls. All procedures were performed according to the previously published protocol, and experiments were repeated to ensure reproducibility. Furthermore, to assess anti-migratory potential of fisetin on HeLa cells, wound healing and transwell assays were carried out following the protocol published earlier. Cells were treated with fisetin at 20, 30, and 50 µM, while untreated cells served as controls. Cell migration was documented at 0 h and every 24 h until the wound was fully closed. Wound widths were determined from the images using MS Paint, and the data were presented as the percentage of wound closure in bar graph format. At the same concentrations, the invasive potential of the cells was assessed based on their ability to traverse the extracellular matrix (ECM), a characteristic feature associated with cancer metastasis. This assay also evaluates the cells’ response to chemoattractant, which in this study was provided by the growth factors present in fetal bovine serum (FBS).

### 4.6. Impact of Fisetin on Protein Expression and the Pathway Associated with Migration

The human MMP antibody array kit (Abcam, Cambridge, UK; Cat No# ab134004) was employed to assess the expression of migration-associated proteins, and evaluation of proteins involved in the TGFβ pathway was performed using the phosphorylation kit of TGFβ pathway from RayBio^®^ (Peachtree Corners, GA, USA). Relative protein expression was assessed according to the instructions provided and following the previously published protocol [47]. The relative expression levels were quantified by evaluating dot-blot intensities on treated vs. control membranes using Image Lab software (version 6.1), with statistical significance at *p* ≤ 0.05.

### 4.7. Analysis of Promoter Methylation Status by Fisetin Through Methyl Sequencing

Methyl-sequencing analysis was performed to investigate DNA CpG methylation and its alteration upon fisetin treatment in HeLa cells. Isolated DNA was evaluated for both quality and concentration using a Nanodrop Spectrophotometer (Thermo Scientific, Waltham, MA, USA, 2000), after which libraries were prepared for sequencing. Detailed methodology was replicated from an earlier published paper [46]. Methylation differences greater than 0.10 with *p*-values less than 0.05 were considered crucial. To visualize differential methylation, a heatmap was created using the gplots R package, (version gplots 3.1.1) for control and fisetin (20 µM)-treated HeLa cells. Gene names corresponding to relevant biological processes were compiled, and the top 40 genes were used to construct networks using the ShinyGO tool.

### 4.8. Global DNA Methylation Assay-Line 1

To examine changes in the methylation of repetitive sequences after fisetin treatment (20–50 µM), the Global DNA Methylation LINE-1 Kit (Active Motif, Carlsbad, CA, USA; Cat No# 55017) was used following the published protocol [46]. Values represent the mean ± SD of three experiments, and differences were considered significant at *p* ≤ 0.05.

### 4.9. Effect of Fisetin on Expression of Selected Genes at MRNA Level

A TaqMan-based custom array(Ap plied Biosystems, Thermo Fisher Scientific, Waltham, MA, USA; Cat. No# 4391594) was employed to measure the expression of genes involved in tumor suppression, migration, and signal transduction in fisetin-treated and control cells. Total RNA was extracted from 50 µM fisetin-treated cells and DMSO controls with the GenElute Mammalian Genomic Total RNA Kit Sigma-Aldrich, Merck KGaA, (St. Louis, MO, USA; Cat. No# RTN70), and cDNA was synthesized as described earlier [8]. Fold changes in gene expression were evaluated using DataAssist™. GAPDH serves as the housekeeping gene for normalization.

### 4.10. Evaluation of Histone H3 and H4 Modifications

Effect of fisetin treatment (50 μM) on histone H4 and H3 modification marks was evaluated with the histone extraction kit (Abcam, Cambridge, UK; Cat. No# ab113476) and H4 and H3 modification multiplex assay kits (Abcam, Cambridge, UK; Cat No# ab185910 and ab185914). The assay was performed following the kit’s protocol as our lab published previously [8]. For all biochemical assays, data were evaluated and significance was set at *p* ≤ 0.05.

### 4.11. Statistical Analysis

The results were reported from three independent experiments (mean ± SD). GraphPad Prism (version 9.3.1) was used to statistically evaluate significance using two-way ANOVA or *t*-tests, followed by Tukey’s HSD post hoc analysis.

## 5. Conclusions

Treatment with fisetin caused pronounced alterations in the expression levels of several epigenetic regulators, including DNMTs, HDACs, HATs, and HMTs, accompanied by hypomethylation of key TSGs. Concordant with transcriptional changes, biochemical assays revealed dose-dependent decreases in DNMT, HAT, HMT, and HDAC activities, suggesting direct enzyme inhibition. Fisetin was well tolerated, as global LINE-1 methylation levels remained unaffected. These epigenetic modifications corresponded with the inhibition of HeLa cell proliferation and migration. To enhance fisetin’s bioavailability, future investigations should consider nanoformulation or pro-drug strategies, followed by in vivo studies to substantiate its safety profile and epigenetic efficacy before clinical translation.

## Figures and Tables

**Table 1:** Amino acid interaction (highlighted as bold, vina score) of fisetin–protein complex and native inhibitor–protein complex.

Proteins/Receptors	Energy/Vina Score	Docking Coordinates (CB Dock)	Interacting Amino Acids with Fisetin (PLIP)	Interacting Amino Acids with Native Inhibitor (PLIP)	Hydrogen Bond Between Fisetin and Protein (PLIP)	Hydrogen Bond Between Native Inhibitor and Protein (PLIP)	π-Stacking Between Fisetin and Protein (PLIP)	π-Stacking Between Native Inhibitor and Protein
DNMT 3 B 6U8V	−9.0	52, −49, 2	PRO 650, **ILE 584**, **THR 586**, GLU 605, CYS 651, **TRP 834**, **ARG 832**	PHE 581, **ILE 584**, **THR 586**, VAL 606, ASP 627, VAL 628, **ARG 832**, **TRP 834**	**ILE 584**, **THR 586**, GLU 605, CYS 651, **TRP 834**	PHE 581, **ILE 584**, **THR 586**, VAL 606, ASP 627, VAL 628, ARG 832, **TRP 834**	TRP 834	PHE 581
HAT 1 9MJG	−7.7	−6, −9, −56	**MET 241**, **ILE 243**, **TYR 282**, **GLN 248**, SER 279	**MET 241**, **TYR 282**, LEU 285, **ILE 243**, **GLN 248**, GLY 249, SER 281	**MET 241**, **ILE 243**, **GLN 248**, SER 279	**MET 241**, **ILE 243**, **GLN 248**, GLY 249, SER 281	NONE	NONE
HMT 1 4HC4	−8.2	23, 45, −5	LEU 162, ARG 351, SER 165, SER 168, LEU 343, LEU 344, ARG 354	HIS 57, GLY 90, GLY 92, ALA 113, SER 114, VAL 140, GLU 141, ARG 141	SER 165, SER 168, LEU 343, LEU 344, ARG 354	HIS 57, GLY 90, GLY 92, ALA 113, SER 114, VAL 140, GLU 141	NONE	NONE
HDAC 8 1T69	−7.2	32, −2, −9	PHE 207, ASP 101, TYR 306, **PHE 152**, **PHE 208**	TYR 100, **PHE 152**, **PHE 208**, HIS 180	ASP 101, TYR306	HIS 180	NONE	NONE

**Table 2:** Table showing inhibition of various histone H3/H4 mark modifications after fisetin treatment. Values represent mean ± SD (*n* = 3). SD ranged from 1.0 to 2.0.

Histone H3 Marks	% Inhibition	Histone H4 Marks	% Inhibition
H3K36me2	32.31	H4K20m2a	18.84
H3K4me3	37.75	H4R3m2s	25.975
H3K9me3	34.885	H4K16ac	28.25
H3K4me2	40.23	H4K20m1	23.385
H3K27me3	46.89	H4K12ac	24.65
H3K9me2	51.325	H4R3m2a	27.235
H3K27me1	54.17	H4K20m3	25.455
H3K9me1	52.595	H4K5ac	25.985
H3ser10ph	84.08	H4ser1	29.39
H3K27me2	55.48		
H3K36me3	58.525		
H3K56ac	60.66		
H3K9ac	61.34		
H3K79me3	70.55		
H3K36me1	65.385		
H3K14ac	68.775		
H3K4me1	71.685		
H3ser28ph	77.58		
H3K18ac	101.89		
H3K79me2	63.99		
H3K79me1	83.08		

## Data Availability

The authors declare that all data supporting the findings of this study are included within the manuscript, and no external datasets were generated or analyzed.
